# What does the scale‐up of long‐acting HIV pre‐exposure prophylaxis mean for the global hepatitis B epidemic?

**DOI:** 10.1002/jia2.26218

**Published:** 2024-03-05

**Authors:** Amir M. Mohareb, Menan Gérard Kouamé, Marcellin Nouaman, Arthur Y. Kim, Joseph Larmarange, Anne M. Neilan, Karine Lacombe, Kenneth A. Freedberg, Anders Boyd, Patrick Coffie, Emily P. Hyle

**Affiliations:** ^1^ Medical Practice Evaluation Center Massachusetts General Hospital Boston Massachusetts USA; ^2^ Division of Infectious Diseases Massachusetts General Hospital Boston Massachusetts USA; ^3^ Harvard University Center for AIDS Research Boston Massachusetts USA; ^4^ Département de Santé Publique UFR d'Odonto‐stomatologie Université Félix Houphouët Boigny Abidjan Côte d'Ivoire; ^5^ Centre Population et Développement Université Paris Cité, IRD, Inserm Paris France; ^6^ Division of General Academic Pediatrics Department of Pediatrics Massachusetts General Hospital Boston Massachusetts USA; ^7^ Sorbonne Université, IPLESP Paris France; ^8^ Department of Infectious Diseases St. Antoine Hospital, AP‐HP Paris France; ^9^ Department of General Internal Medicine Massachusetts General Hospital Boston Massachusetts USA; ^10^ Stichting HIV Monitoring Amsterdam the Netherlands; ^11^ Department of Infectious Diseases Public Health Service of Amsterdam Amsterdam the Netherlands; ^12^ Amsterdam UMC, Infectious Diseases Amsterdam the Netherlands; ^13^ Amsterdam Institute for Infection and Immunity, Infectious Diseases Amsterdam the Netherlands; ^14^ Département de Dermatologie et Infectiologie Université Félix Houphouët‐Boigny Abidjan Côte d'Ivoire

**Keywords:** cabotegravir, hepatitis B, hepatitis, HIV, long‐acting antiretroviral therapy, long‐acting PrEP, PrEP, tenofovir

## Abstract

**Introduction:**

The HIV and hepatitis B virus (HBV) epidemics are interconnected with shared routes of transmission and specific antiviral drugs that are effective against both viruses. Nearly, 300 million people around the world live with chronic HBV, many of whom are from priority populations who could benefit from HIV prevention services. Oral pre‐exposure prophylaxis (PrEP) for HIV has implications in the prevention and treatment of HBV infection, but many people at increased risk of HIV acquisition may instead prefer long‐acting formulations of PrEP, which are currently not active against HBV.

**Discussion:**

People at increased risk for HIV acquisition may also be at risk for or already be living with HBV infection. Oral PrEP with tenofovir is effective in preventing both HIV and HBV, and tenofovir is also the recommended treatment for chronic HBV infection. Although implementation of oral PrEP has been challenging in sub‐Saharan Africa, investments in its scale‐up could secondarily reduce the clinical impact of HBV. Long‐acting PrEP, including injectable medicines and implantable rings, may overcome some of the implementation challenges associated with oral PrEP, such as daily pill burden, adherence challenges and stigma; however, current formulations of long‐acting PrEP do not have activity against HBV replication. Ideally, PrEP programmes would offer both oral and long‐acting formulations with HBV screening to optimize HIV prevention services and HBV prevention and care, when appropriate. People who are not immune to HBV would benefit from being vaccinated against HBV before initiating long‐acting PrEP. People who remain non‐immune to HBV despite vaccination may benefit from being offered oral, tenofovir‐based PrEP given its potential for HBV PrEP. People using PrEP and living with HBV who are not linked to dedicated HBV care would also benefit from laboratory monitoring at PrEP sites to ensure safety when using and after stopping tenofovir. PrEP programmes are ideal venues to offer HBV screening, HBV vaccination for people who are non‐immune and treatment with tenofovir‐based PrEP for people with indications for HBV therapy.

**Conclusions:**

Long‐acting PrEP holds promise for reducing HIV incidence, but its implications for the HBV epidemic, particularly in sub‐Saharan Africa, should not be overlooked.

## 1 INTRODUCTION

The HIV and hepatitis B virus (HBV) epidemics are interconnected [1]. The two viruses share many of the same routes of transmission and both benefit from focused prevention efforts that reduce exposures to blood‐borne and sexually transmitted pathogens [1, 2]. People at increased risk for HIV and HBV acquisition also benefit from behaviour modifications aimed at prevention, such as the use of condoms during sex, needle exchange programmes for injection drug use, blood donor screening and prevention of needlestick injury [3]. HIV and HBV are also both treated by some of the same first‐line antiretroviral therapy (ART), namely, tenofovir disoproxil fumarate or tenofovir alafenamide and lamivudine. Prevention of HIV and HBV acquisition individually are long‐standing public health priorities, and prevention of HIV‐HBV coinfection is particularly important because of the added risks of coinfection, including increased risks of liver disease, cancer and all‐cause mortality even when people are treated with dually active ART [4, 5, 6].

Nearly, 300 million people around the world live with chronic HBV, approximately 10 times the number with HIV, and 1.5 million new HBV infections occur each year [3, 7]. HBV is the leading global cause of chronic liver disease and liver cancer. HBV is endemic in sub‐Saharan Africa (prevalence of chronic infection >7.0%), the Western Pacific (5.7%) and Southeast Asia (3.5%) [7]. In non‐endemic settings, many key populations have an even higher prevalence of chronic HBV: estimated HBV prevalence among people who use drugs in Europe is as high as 17% in some studies [8]. In many studies, men appear to have more persistent HBV infection and a higher incidence of fibrosis and hepatocellular carcinoma than women [9]. Most PrEP trials excluded people living with HBV from participation, which has limited the available data on the impact of PrEP on HBV disease progression [10]. There are no systematic reviews evaluating the prevalence of HBV among people taking PrEP, but the same risk factors for HIV acquisition may also increase the risk of acquisition of HBV and other blood‐borne and sexually transmitted infections.

Oral HIV pre‐exposure prophylaxis (PrEP) with tenofovir and emtricitabine has the potential to prevent new HIV acquisitions in key populations, but implementation challenges have limited the global impact of PrEP thus far [11]. A combination of factors have slowed the rollout of PrEP in low‐ and middle‐income countries, including funding limitations and the lack of tailored adherence support [11, 12]. Key populations, including men who have sex with men, people who sell sex and people who use injection drugs, face a variety of structural barriers, such as discrimination, stigma, criminalization and violence, which further limit access to preventive care in nearly all settings [13]. The recent development of long‐acting PrEP could address some of these barriers, given the high acceptability and preference for less frequently dosed, long‐acting modalities that are safe and effective at preventing HIV [14, 15, 16].

Tenofovir is effective in preventing HBV acquisition [17, 18, 19] and in suppressing HBV replication [20]. A recent study in Japan demonstrated that men who have sex with men who used daily or event‐driven oral PrEP had a dramatic reduction in their risk of HBV acquisition compared with non‐users: no HBV infections among people adherent to PrEP compared with 1.8 infections per 100 person‐years in people who did not use PrEP [19]. For people with chronic HBV, cessation of long‐term tenofovir therapy introduces the risk of hepatitis flares, which can complicate PrEP discontinuation [20]. In sub‐Saharan Africa, the costs of HBV monitoring and treatment with tenofovir are most often borne by individuals [9, 21], which make these life‐saving interventions inaccessible to most people living with HBV in this region. HIV prevention programmes could make important gains in the global HBV elimination effort through expanded HBV screening and access to tenofovir therapy for key populations through programmes that offer oral PrEP [20, 22]. However, currently available formulations of long‐acting PrEP, like injectable cabotegravir, are not active against HBV [23], which introduces a challenge in HBV‐endemic settings where long‐acting PrEP formulations are anticipated to be scaled up [24]. Therefore, the benefits of long‐acting PrEP for improved adherence and more widespread use should be evaluated along with its current limitations in contributing to the global HBV elimination effort.

## 2 DISCUSSION

For many people at increased risk of HIV acquisition, a cornerstone of HIV prevention is pharmacologic PrEP [25]. New HIV infections disproportionately impact key populations who face structural barriers to equitable care in nearly all health systems around the world [13]. Oral PrEP with tenofovir‐based regimens reduces the risk of HIV by more than 90% in randomized controlled trials of men who have sex with men and serodiscordant couples [26]. Similar results have not been seen for women living with HIV, and some PrEP strategies, such as event‐based PrEP, are not recommended in women [27, 28]. Event‐based PrEP may also have limitations in its implementation for some people, as a recent multi‐country cohort in West Africa demonstrated a higher HIV incidence rate (2.4 per 100 person‐years) among men who have sex with men and chose event‐based PrEP compared with those who chose daily PrEP (0.6 per 100 person‐years) [29]. Efforts at scaling up PrEP among key populations have encountered many obstacles, including financial barriers related to drug and programme costs, low public awareness of the benefits of PrEP, low self‐perceived HIV risk and healthcare capacity challenges in low‐ and middle‐income health systems [11].

There are several important considerations for the use of oral PrEP among people living with HBV [20, 30]. People who would benefit from PrEP would also benefit from the treatment of chronic HBV [31]. Somewhat fortuitously, the same antiviral drugs that form the basis of oral PrEP—tenofovir disoproxil fumarate or tenofovir alafenamide with emtricitabine—are also potent inhibitors of HBV polymerase. People living with HBV and chronic liver disease may see regression of fibrosis after years of treatment with tenofovir [32]; tenofovir treatment reduces the risk of cirrhosis and hepatocellular carcinoma in people with HBV‐related hepatitis [33]. This means that oral, tenofovir‐based PrEP has the potential to simultaneously prevent HIV and treat chronic, active HBV [20]. The ongoing scale‐up of PrEP programmes around the world, therefore, represents an important public health opportunity to improve HBV care, both by expanding HBV screening, which is recommended before or at the initiation of PrEP [34], and by increasing access to HBV‐active antiviral therapy for people living with chronic HBV who meet criteria for HBV treatment or are PrEP‐eligible.

Just as PrEP scale‐up has faced implementation challenges, so too have the efforts to expand HBV screening and treatment in resource‐limited settings [35]. Of the more than 70 million people living with HBV in sub‐Saharan Africa, less than 5% are aware of their diagnosis, and less than 1% of treatment‐eligible people are prescribed HBV‐active antivirals [7]. Since most countries in sub‐Saharan Africa lack comprehensive HBV management programmes, the costs for HBV screening, monitoring and treatment are usually shouldered by individuals and their families [21, 36]. Unlike HIV, only a minority of people living with chronic HBV have indications for treatment [30]. Establishing the clinical criteria for treatment eligibility in each person with HBV is a complex process, even in the most comprehensive health systems, because it relies on repeatedly assessing both liver fibrosis (e.g. transient liver elastography or liver biopsy) and HBV viral markers (e.g. HBV “e” antigen and HBV DNA) [30]. In many parts of the world, these diagnostic tests for monitoring HBV are inaccessible [36, 37].

Growing attention and resources devoted to PrEP scale‐up in resource‐limited settings can have a positive impact on the global HBV elimination campaign by serving as an opportunity for HBV screening, HBV prevention and an access point for routine monitoring during antiviral therapy. PrEP sites can offer HBV prevention with HBV vaccination; the typical HBV vaccine schedule (three doses at 0, 1 and 6 months) can be adapted to coincide with return visits for PrEP (e.g. 0, 3 and 9 months for oral PrEP and 0, 2 and 8 months for injectable cabotegravir) without diminishing vaccine efficacy [38]. People who remain non‐immune to HBV following vaccination would benefit from regular screening for HBV acquisition, consistent with World Health Organization (WHO) guidelines for screening sexually transmitted infections among people engaged in PrEP care [34]. HBV screening using point‐of‐care testing is an accurate and feasible approach to conduct community‐based screening at low cost [39, 40, 41]. However, at the current time, most HIV PrEP and HBV treatment programmes in sub‐Saharan Africa are separate, funded through different mechanisms and implemented at different clinical sites [9, 21]. Settings well‐equipped with HIV PrEP and HIV care providers are often lacking in clinicians experienced in HBV care [9].

While tenofovir‐based PrEP provides an opportunity to engage people in longer‐term HBV care, it also comes with unique risks for people living with HBV [22]. Event‐based PrEP (i.e. planned use of PrEP around the time of condomless sex) would be unlikely to yield substantial benefits in HBV DNA suppression owing to the inconsistent use of therapy, although it can still be effective against HIV transmission [34]. Importantly, people who have suppressed HBV DNA while on long‐term antiviral therapy may develop HBV virologic relapse (i.e. an acute rise in HBV DNA) and a potentially life‐threatening hepatitis flare if antivirals are stopped [42]. This has been demonstrated in a number of settings, ranging from short‐term antiviral use in people with HBV who are pregnant to controlled studies of antiviral treatment cessation in HBV monoinfection, where virologic relapse occurs in up to 65% of people after stopping HBV antivirals [10, 43–46].

Observational clinical data of hepatitis flares in the context of PrEP cessation are still lacking. The WHO now emphasizes that the “lack of HBV testing should not be a barrier to PrEP initiation or use,” despite the “small risk of HBV relapse” following PrEP cessation in people with chronic HBV [34]. Many people who use oral PrEP face challenges with adherence and access, or they may elect to use PrEP only around the time of condomless sex. Tenofovir use for days or weeks by people with HBV is unlikely to cause hepatitis flares given limited effects on HBV DNA levels with short‐term use, but cessation after longer periods of adherence may result in a clinically significant hepatitis flare [10, 43, 44]. These considerations associated with antiviral cessation can be safely managed by PrEP prescribers by, first, screening PrEP users for HBV as recommended by the WHO to identify who is at risk for HBV virologic relapse and, second, by close monitoring of HBV activity when PrEP is stopped and resumption of HBV‐active antiviral therapy in cases of severe hepatitis flares. Post‐cessation monitoring is, however, complex, and settings that lack HBV DNA testing might need to rely on serial testing of aminotransferase levels or forthcoming point‐of‐care testing following PrEP cessation.

As oral PrEP continues to be scaled up, scientific advancements in long‐acting regimens of HIV PrEP are heralding a new era in HIV prevention that overcomes many logistical, adherence and stigma‐related barriers associated with daily oral medication use [16]. The four types of long‐acting PrEP formulations include injectable drugs, intravaginal rings, implantable depots and monoclonal antibodies [24]. Among the most studied of these drugs is cabotegravir, an injectable HIV integrase strand transfer inhibitor that has nearly 90% efficacy in HIV prevention when delivered once every 8 weeks [16, 47]. Concerns regarding long‐acting PrEP include injection site reactions, management of drug−drug interactions with a drug that remains in the body for several weeks and the risk of HIV drug resistance if breakthrough infections occur. In the 10 years since oral PrEP has been available, the full potential of PrEP has still not yet been realized, and many are hoping that long‐acting PrEP will fill some of the current gaps in HIV prevention [12].

Despite its favourable characteristics for improving PrEP uptake and adherence, long‐acting PrEP introduces several new concerns around the global effort to eliminate HBV [23]. Currently available regimens of long‐acting PrEP do not have antiviral activity against HBV [23]. Therefore, these agents do not provide the same opportunity for HBV prevention and treatment as oral, tenofovir‐based PrEP. On the other hand, long‐acting PrEP could be a preferable option for people with chronic HBV at high risk for intermittent or episodic tenofovir use who would be at increased risk of hepatitis flare. For HBV‐endemic settings, emphasis on long‐acting PrEP at the expense of oral PrEP would be a lost opportunity to expand HBV screening and treatment because PrEP programmes are ideally situated as sites to deliver HBV screening, monitoring and antiviral therapy, particularly for key populations. At the same time, having the option of long‐acting PrEP would make available an alternative means of HIV prevention that could be more desirable to some. The imminent global scale‐up of long‐acting PrEP may, therefore, introduce a dialectic between two different public health needs: decreasing the burden of HBV through expanded screening and antiviral access and preventing HIV transmission more effectively. The expansion of PrEP programmes can most effectively address HIV and HBV burden if they include both oral and long‐acting PrEP formulations and HBV screening, HBV vaccination and management of active HBV infection using laboratory monitoring and oral, tenofovir‐based PrEP (Table 1).

**Table 1:** HIV and hepatitis B virus considerations with the scale‐up of pre‐exposure prophylaxis programmes for key populations.

	Challenges and considerations	Role for PrEP programmes
**Preventing HIV**	Adherence challenges and stigma associated with daily medication use	Include long‐acting options for PrEP
	Prevention counselling and adherence support	Strengthen healthcare workers capacity and training
	Safety and monitoring of PrEP adverse effects	Routine laboratory monitoring for PrEP users
	Rapid diagnosis of HIV with linkage to HIV care among PrEP users to avoid development of resistance	Ensure PrEP programmes have standard approaches to diagnosis of incident HIV and linkage to HIV care
	Screening and linkage to care for HBV and STIs	Equip PrEP programmes with diagnostic capacity for routine testing for HBV and other STIs
**Preventing acute HBV**	Prevention of HBV acquisition for people who are non‐immune	HBV vaccination and/or use of tenofovir‐based PrEP
	Prevention of HBV infection for people who do not respond to HBV vaccination	Use of oral tenofovir‐based PrEP
**Treating HBV**	Screening and diagnosis of HBV	HBV screening at or close to initiation of PrEP
	Monitoring for HBV‐related liver complications (i.e. cirrhosis and HCC)	Equip PrEP programmes with the ability to test and interpret results for non‐invasive markers of liver disease, or link to care sites with that capacity
	Access to antiviral therapy for people with chronic HBV and indications for antiviral treatment	Include oral, tenofovir‐based PrEP for dual HIV prevention and chronic HBV treatment
	Risk of HBV reactivation if antiviral therapy is stopped	Screening for chronic HBV and monitoring following PrEP cessation for people living with HBV
	Clinical complexity in determining HBV treatment eligibility	Simplify HBV treatment initiation criteria or expand access to testing needed to ascertain HBV treatment eligibility
	Costs of chronic HBV diagnosis and treatment	Budget for HBV testing and treatment in PrEP programmes

Abbreviations: HBV, hepatitis B virus; HCC, hepatocellular carcinoma; PrEP, pre‐exposure prophylaxis; STI, sexually transmitted infection.

In light of the advancing science and anticipated expansion and availability of long‐acting PrEP formulations, the following recommendations could ensure equitable inclusion of people living with HBV into HIV prevention efforts (Table 1). First, PrEP programmes should strive to include both long‐acting and oral, tenofovir‐based formulations, so they can adapt to a diversity of individual needs. This will mean equipping programmes, particularly in low‐ and middle‐income countries, with the finances and staffing to deliver different formulations of PrEP with person‐centred counselling. Second, clinical implementation of long‐acting PrEP should also include HBV screening so that people are best informed about their options for PrEP. At a minimum, HBV screening should include testing for HBsAg and ideally HBV core and surface antibodies to identify who is non‐immune. Testing for HBV should not delay initiation of oral or long‐acting PrEP; incorporating HBV testing will best serve the needs of people who are eligible for PrEP by identifying people who would benefit either from antiviral therapy if they have HBV infection or from HBV vaccination if they are non‐immune. The prevalence of HBV infection among PrEP users likely varies between settings and might have sex and gender differences; an improved understanding of the number of people who have indications for both HBV treatment and PrEP will be important for programme planning and implementation. Finally, research in long‐acting PrEP formulations should prioritize the incorporation of HBV‐active antiviral therapies so that people at risk of or living with HIV‐HBV coinfection can benefit from the advancing science of long‐acting drug delivery [23, 48].

## 3 CONCLUSIONS

Through their shared susceptibility to tenofovir, the HIV and HBV epidemics can both benefit from the scale‐up of oral PrEP. Long‐acting PrEP undoubtedly holds much promise for overcoming the current barriers in reducing HIV incidence, but its implications for the HBV epidemic, particularly in sub‐Saharan Africa, should not be overlooked. While long‐acting PrEP could improve HIV prevention efforts, it should not supplant the use of oral, tenofovir‐based PrEP for people with indications for HBV treatment. PrEP programmes should emphasize HBV screening, HBV vaccination, include both long‐acting and oral, tenofovir‐based formulations of PrEP, and incorporate monitoring for HBV disease during routine PrEP care. While this approach will require additional investments for drug availability, counselling and clinical capacity, these efforts are critical to improve prevention and treatment for both HIV and HBV.

## COMPETING INTERESTS

KL reports grants from Gilead, Janssen, Roche and ViiV. The other authors declare no competing interests.

## AUTHORS’ CONTRIBUTIONS

AMM and EPH conceptualized the article. AMM primarily drafted the article. All authors reviewed the manuscript and edited it for important intellectual content. All authors approved of the final version of the manuscript for submission.

## FUNDING

AMM is supported by the National Institute for Allergy and Infectious Diseases (NIAID) at the National Institutes of Health (NIH) (grant number K01AI166126) and the Harvard University Center for AIDS Research (grant number P30AI060354). AMN is supported by the Eunice Kennedy Shriver National Institute of Child Health and Human Development at the NIH (grant number R01HD111355) and the Massachusetts General Hospital (MGH) Department of Medicine Transformative Scholars Award. KAF is supported by NIAID at the NIH (grant number R37AI058736). EPH is supported by the Jerome and Celia Reich Endowed Scholar in HIV/AIDS Research at MGH.

## DISCLAIMER

The funders had no role in the design or authorship of this publication. The article contents are solely the responsibility of the authors and do not necessarily represent the official views of the funders.

## Data Availability

The data that support the findings of this Commentary are available on request from the corresponding author.
